# Cryostructuring of Polymeric Systems: 68. Evaluation of Poly(vinyl alcohol) Composite Cryogels Filled with Poly(3-hydroxybutyric acid)-Based Microspheres of Different Porous Morphology as Potential Delivery Systems for Drugs of Various Water-Solubility †

**DOI:** 10.3390/gels10110734

**Published:** 2024-11-13

**Authors:** Dmitrii A. Michurov, Gagik A. Andreasyan, Vladimir I. Lozinsky

**Affiliations:** 1 A.N. Nesmeyanov Institute of Organoelement Compounds, Russian Academy of Sciences, Vavilov Street 28, Building 1, 119334 Moscow, Russia; dmitriial7.8@gmail.com (D.A.M.); futureinthepasttt@gmail.com (G.A.A.); 2 Microbiology Department, Kazan (Volga-Region) Federal University, 420008 Kazan, Russia

**Keywords:** poly(vinyl alcohol), poly(3-hydroxybutyric acid), porous microspheres, composite cryogels, physico-mechanical properties, drug release, simvastatin, ibuprofen sodium salt

## Abstract

Poly(3-hydroxybutyric acid)-based microspheres of two types, with and without macropores, were prepared; their morphology and particle size were evaluated. These microspheres were entrapped as disperse fillers into the bulk of macroporous cryogels based on poly(vinyl alcohol) (PVA). It was found that the rigidity of the resultant composite cryogels increased markedly as compared to that of unfilled cryogels of the same PVA concentration. The resulting composites were further tested for their potential to act as drug carriers. With that, simvastatin was included into the filler particles directly in the course of their preparation, followed by entrapment of such drug-loaded microspheres into the PVA cryogel. In turn, ibuprofen sodium salt was introduced into the preliminary prepared cryogels filled with the drug-free microspheres. The experimental study of drug release kinetics showed that due to the non-covalent interactions of both simvastatin and ibuprofen sodium salt with the particles of discrete phase, prolongation of the release processes was observed.

## 1. Introduction

At present, the interdisciplinary R&D related to the polymeric gel systems intended for biomedical applications is intensively progressing [1,2,3]. Among various gel materials are the physical poly(vinyl alcohol)-based cryogels (PVACGs) that are formed as a result of consecutive freezing of concentrated solutions of highly deacylated poly(vinyl alcohol) samples, their incubation in a frozen state, and final defrosting [4,5,6] These macroporous gel matrices attract marked interest in the biomedical area [7,8,9,10,11,12,13,14,15] since PVACGs are biocompatible [10,16], non-toxic [17] and non-carcinogenic polymeric materials [7,10] also possessing excellent diffusion [7,8,9] and physicomechanical properties [4,5,6,18,19,20,21]. Also, there are wide opportunities to vary these properties, including the preparation of composite PVACGs containing appropriate functional disperse fillers [22,23,24]. Additionally, PVA cryogels of various geometric shape and size can be easily fabricated. For this reason, numerous promising materials of biomedical interest have been elaborated, in particular, drug delivery systems [8,9,10], therapeutic covers on wounds [11,12], biomimetic prostheses [13,14,15], spherical particles [25], temporary implants [26], etc.

The examples of composite PVACGs that contained disperse fillers (Na-montmorillonite, calcium phosphate, kaolin, PLGA nanoparticles, chitosan-based microspheres, etc.) have also been reported and developed for biomedical applications (see Refs. [22,23,24,27,28,29]). It was shown that disperse fillers of various chemical nature, a broad range of particles’ size, and relatively simple methods for preparing the required fillers can be used [30].

It is known that various fillers can generally be classified as follows: (i) inert fillers that do not significantly affect the properties of the matrix, (ii) so-called ‘active’ fillers that improve the physicomechanical characteristics of the respective composites, and (iii) the fillers that are able to worsen the rigidity of composite materials [31]. The filler activity is mainly conditioned by such factors as the adhesion energy of the polymer to the filler, the cohesion energy of the polymer, the size of the filler particles (it determines the contact surface area between the matrix and the filler), the amount of the introduced filler [32]. In the case of biomedical composite materials, including the drug delivery systems, various microspheres were used as the fillers due to the possibility to control the release rate, thus reducing the dose of the respective medication [27,33].

In the previous study, we prepared and examined some properties of composite PVA cryogels filled with macroporous microspheres based on poly(3-hydroxybutyric acid) (PHB) [34]. With that, the possibility for loading and release of simvastatin into/from the filler particles was also demonstrated. The use of PHB was based on the fact that this microbiologically produced biopolymer has a number of advantages over chemically synthesized polyesters in terms of biocompatibility [35] and biodegradability, resulting in the formation of nontoxic metabolites [36]. In addition, good mechanical properties of the PHB-based materials were also reported [37,38,39,40].

Therefore, it was of interest to elucidate the influence of the type of the PHB microspheres on the properties of the respective PVA–cryogel composites and to evaluate their potential to perform as carriers for drugs that possess different solubility in water. These tasks were the aim of the present study, in which the PHB-based microspheres with (MS-1) and without (MS-2) macropores were used as disperse fillers entrapped into the matrix of PVACG. In doing so, two substances of different lipophilicity, namely ibuprofen sodium salt (Na-IBPF) and simvastatin (SVN) were used as the model drugs.

## 2. Results and Discussion

### 2.1. PHB-Based Microspheres

**Figure 1:**
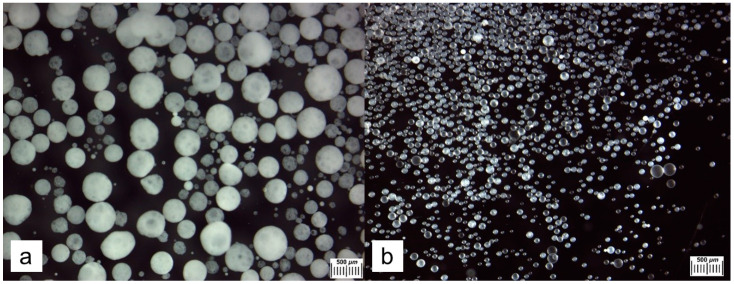
Images (optical stereomicroscope) of wet PHB microspheres: MS-1 (**a**) and MS-2 (**b**).

**Figure 2:**
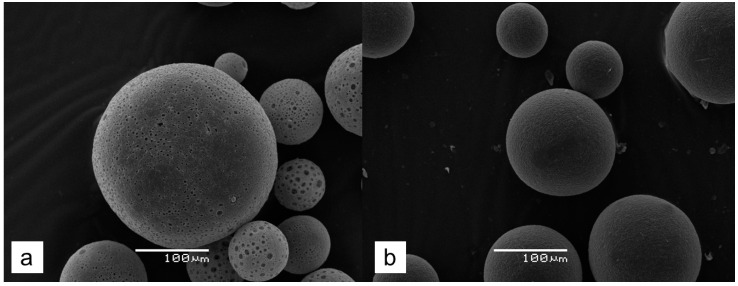
SEM images of dry PHB microspheres: MS-1 (**a**) and MS-2 (**b**).

Two types of PHB-based microspheres were prepared, namely MS-1, which contained macroscopic pores, and MS-2, which did not contain such pores (see Section 4.2.1). These microspheres were either drug-free or loaded with SVN. Then, the respective microbeads were used as fillers for the fabrication of composite PVACGs. The appearance of both MS-1 and MS-2 microspheres in the dry state is shown in Figure 1 (optical microscopy) and Figure 2 (SEM-images).

Numerous rounded cavities (pores) with a diameter of 2–20 μm are observed on the surface of the MS-1 matter (Figure 2a). Such macropores are known [41] to be formed as a result of the entrapment of small drops of the aqueous phase into the organic phase (solution of PHB in chloroform) during emulsification of the PHB/chloroform solution in aqueous solution of ammonium carbonate. In the case of the absence of ammonium carbonate in the aqueous phase, the resulting PHB microspheres did not contain “so large” pores (Figure 2b). The presence of macroscopic pores in the bulk of MS-1 particles increases their inner surface, thus increasing their absorption capacity toward relatively hydrophobic substances. At the same time, the absence of macropores, in addition to decreasing the amount of the loaded substance in the microspheres, could influence the kinetics of the release of substances from MS-2, slowing down the rate of release for drugs entrapped into such particles.

**Figure 3:**
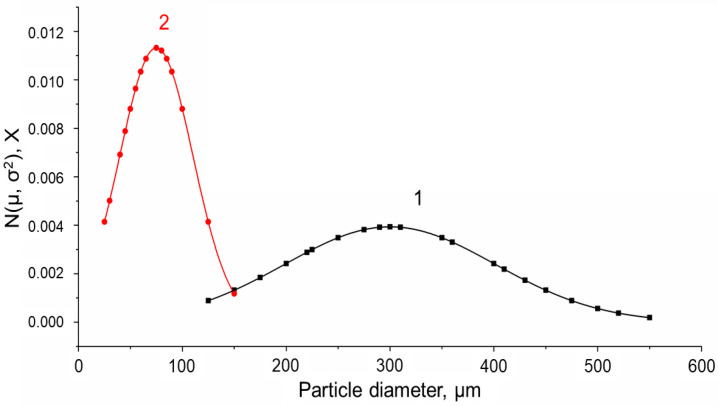
PHB particle size distribution. Curve 1 corresponds to MS-1, curve 2 corresponds to MS-2.

The size of MS-1 beads (Figure 1a and Figure 2a) was several times larger compared to that of the MS-2 particles (Figure 1b and Figure 2b). The images obtained with an optical stereomicroscope (Figure 1) were analyzed using an Image J program. Figure 3 shows the Gaussian distribution of particle diameters (µ). The respective values of the Gaussian function coefficients, mathematical expectation (σ), and standard deviation (R^2^) are presented in Table 1.

It was found that the average diameter of MS-1 was 299 µm and of MS-2 was 75 µm. The sizes of MS-2 were within a narrower range, while the diameters of MS-1 had a much larger deviation. Therefore, at the same mass of spherical PHB particles, the number of microspheres without macroscopic pores, i.e., MS-2 matter, will be greater than the number of MS-1 particles with macroscopic pores.

### 2.2. Physico-Mechanical Properties of the Filler-Free and Composite PVA Cryogels

Physico-mechanical characteristics of cryogels are important parameters of these materials. It is possible to tailor the gel strength of PVACGs for the imitation of a wide range of biological soft tissues [6,10]. For this reason, such materials are attractive for application as prosthetic cartilages [13,14,15], blood vessels [42], heart valves [43], and various implants [10,26].

**Figure 4:**
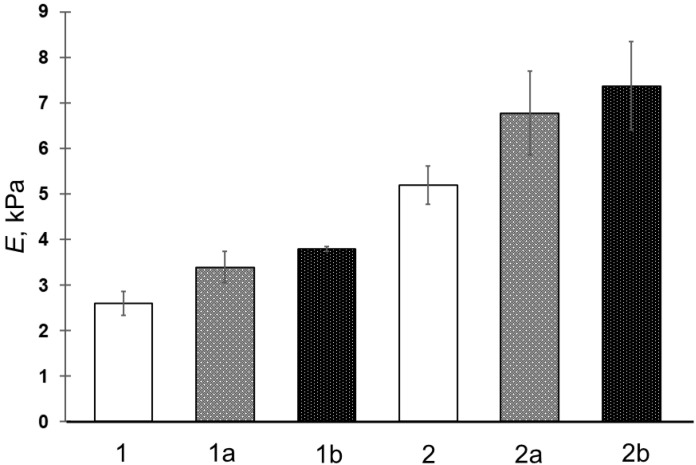
Values of the Young’s modulus of the PVA cryogel samples prepared from the feed compositions indicated in Table 2.

In the present study, upon the formation of composite cryogels, the concentration of the gel-forming polymer, i.e., PVA, was varied, while the mass of PHB-based particles entrapped into the cryogel bulk was identical for all types of microspheres. It was found earlier [34] that when wet PHB-based microspheres are dispersed in the initial solutions of PVA, their distribution over the cryogel matter after its formation is sufficiently uniform; therefore, wet microspheres of preliminary measured moisture content were used. Moreover, the absence of particle cohesion in the initial suspension can also indicate that the distribution of dispersed particles in the gel matrix could be without the presence of some agglomerates of the respective microspheres. It is clear that this assumption requires experimental confirmation, which requires appropriate time and the use of independent methods for the elucidation of the micro- and macrostructure of the composite cryogels under discussion. We have already begun such studies, and their results will be published. The compositions of the corresponding feed solutions and suspensions are given in Table 2, and the compression moduli of elasticity (E) of the respective filler-free and composite PVACGs are illustrated by the diagrams in Figure 4.

The growth of the rigidity of filler-free PVA cryogels with increasing concentrations of the gel-forming polymer is well known [4]. In turn, the ability of some fillers to increase the gel strength of PVACGs indicates that such dispersed fillers, the PHB microspheres in our case (Figure 4), act as so-called “active” fillers [44]. This effect is associated with the higher rigidity of the particles of the discrete phase compared to the continuous phase, and the effect is also provided by good compatibility of these two phases [45]. Thus, the data obtained in the present study testify that the entrapment of relatively hydrophobic PHB microspheres into the matrix of PVA cryogels did not cause deterioration in the mechanical properties of the resultant composite cryogels. Therefore, the use of MS-2 had a somewhat greater effect on the elastic modulus of the composite samples compared to that of the MS-1-filled PVACGs, which is probably due to the difference in the size of filler microbeads and, accordingly, the number of particles per unit volume of PVA cryogel, thus, in the case of MS-2 filler, increasing the contact area of the matrix with dispersed particles.

### 2.3. Drug Release from Unfilled and Composite PVA Cryogels

**Figure 5:**
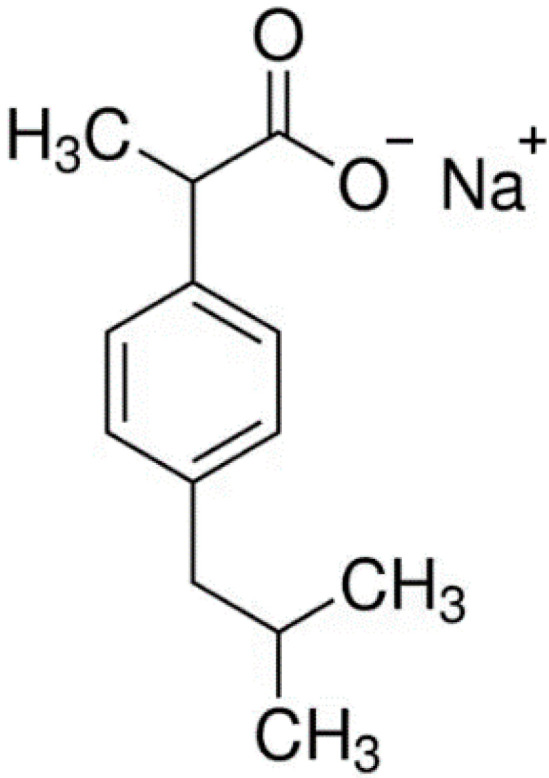
Chemical structure of ibuprofen sodium salt [53].

**Figure 6:**
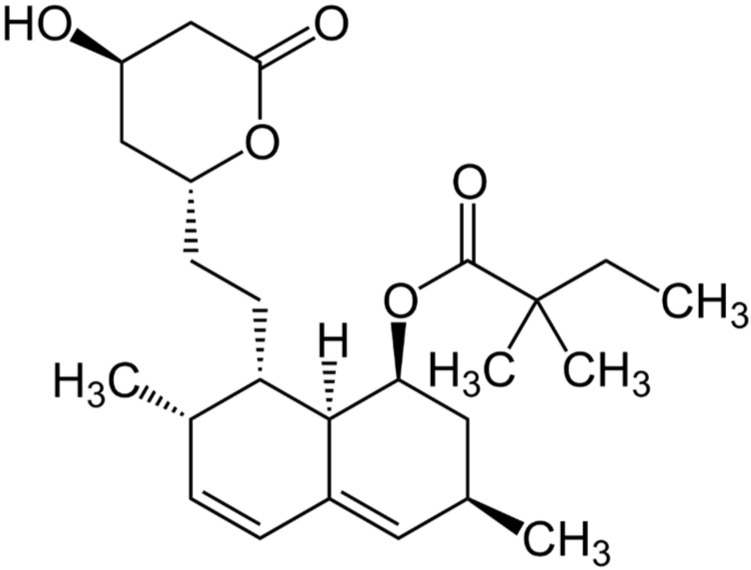
Chemical structure of simvastatin [54].

Ibuprofen sodium salt (Figure 5) and simvastatin (Figure 6) were used as drug compounds in this study. There are publications describing different cryogels as carriers of ibuprofen (including salt form) [46,47] and simvastatin [48,49,50]. In our case, selection of these drugs was made due to the ease of spectrophotometric detecting of both compounds and their different lipophilicity. The latter property can be quantitatively characterized by the distribution coefficient of a substance between two solvents. When one solvent is water and the other is nonpolar liquid, a LogP value serves as a measure of lipophilicity. Consequently, ibuprofen sodium salt is a relatively hydrophilic compound (LogP~0.92) [51], while simvastatin is hydrophobic substance (LogP~4.68) [52]. The drug release study was performed using cryogel samples formed from the starting systems 2, 2a, and 2b (Table 2).

### 2.4. Lease of Ibuprofen Sodium Salt

**Figure 7:**
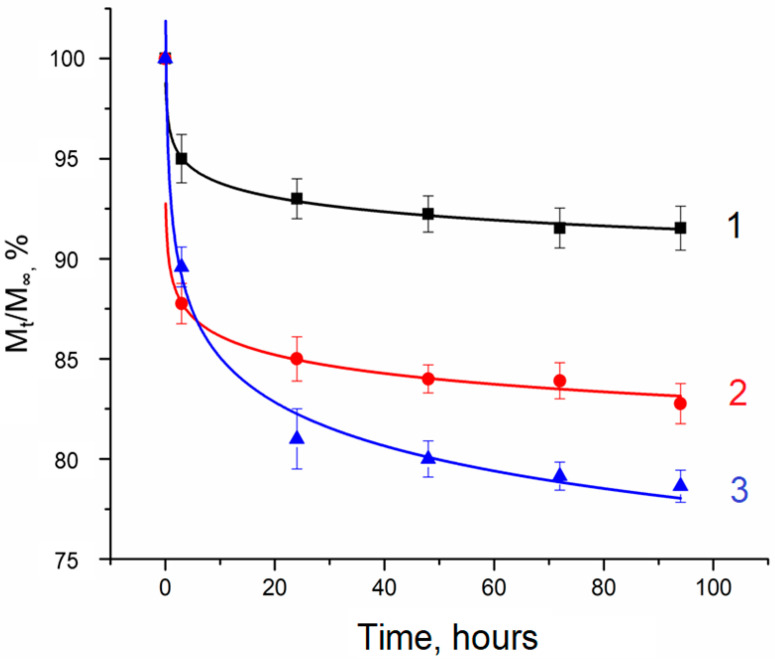
Change in the amount of ibuprofen sodium salt in solution during saturation of samples. Curve 1—cryogel sample without filler; curve 2—cryogel sample with additions of porous PHB microspheres; curve 3—cryogel sample with additions of non-porous PHB microspheres. The mass content of PHB particles in cryogels is equal to ~27 mg/cm^3^ in all cases.

In contrast to simvastatin, which was incorporated into PHB microspheres during their formation, the sodium salt of ibuprofen was introduced into the already-formed cryogel samples. This approach was motivated by the observation that only a small fraction of ibuprofen sodium salt remained in the particles when introduced during the process of microsphere formation (~1–3% for MS-1 and ~17–19% for MS-2). This “loss” is attributed to the leaching of ibuprofen sodium salt at various stages of PHB particle formation. Therefore, composite cryogel samples were saturated with solutions of this drug within a certain time (Figure 7).

The amount of Na-IBPF incorporated into the gel matrix was determined by measuring the optical absorption of the liquid overlaying the cryogel. Knowing the initial concentrations of the solutions and the concentration during and after sample saturation, the amount of ibuprofen sodium salt in the cryogels was quantified (Figure 7). Notably, the amount of drug in solution ceased to change significantly after approximately 24 h. The ~2.1 mL PVA cryogel samples without filler absorbed a smaller amount of Na-IBPF (~0.375 mg) compared to composite samples (~0.75 mg for samples filled with MS-1 and ~1 mg for those filled with MS-2). Different drug uptakes by composite cryogels and unfilled PVA cryogels arise from the presence of PHB microspheres, which facilitate drug absorption. Furthermore, the difference between the composite cryogels that contained MS-1 and MS-2 is attributable to the larger surface area of MS-2, which enhances drug absorption. The greater amount of non-porous microspheres per unit volume of the composite also increases the interaction area, providing greater drug binding.

**Figure 8:**
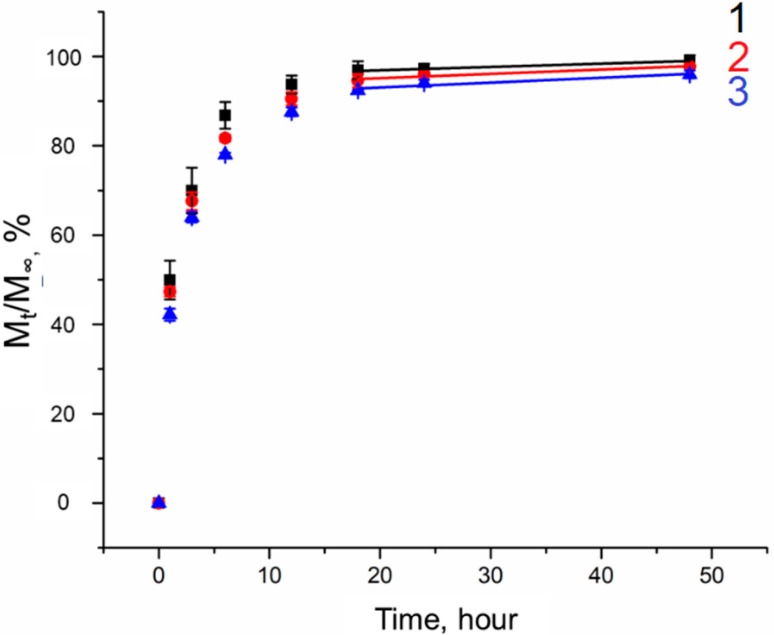
Ibuprofen sodium salt release. Curve 1—cryogel sample without filler; curve 2—cryogel sample with the addition of porous PHB microspheres; curve 3—cryogel sample with the addition of non-porous PHB microspheres.

Figure 8 presents a plot of Na-IBPF release from PVA cryogels. The kinetics of drug release from loaded cryogel samples was analyzed using first-order (1), Higuchi (2), and Peppas–Korsmeyer (3) models, applied to the nonlinear portions of the curves.

ln(M_t_/M_∞_) = k_1_t, (1)

where M_t_ is the amount of drug released at the time t, M_∞_ is the drug amount released at the infinite time, and k_1_ is the first-order constant [55].

M_t_/M_∞_ = k_h_√t, (2)

where M_t_ is the amount of drug released at the time t, M_∞_ is the drug amount released at the infinite time, and k_h_ is the release constant of Higuchi [55,56].

M_t_/M_∞_ = Kt^n^, (3)

where M_t_/M_∞_ is the fraction of drug released at each time point (t), K is the constant of incorporation of structural modifications and geometrical characteristics of the system (also considered the release velocity constant), and n is the exponent of release (related to the drug release mechanism) in function of time. For cylindrical samples, n ≤ 0.45—Fickian diffusion; n > 0.45—non-Fickian mechanisms (anomalous transport) [55,57].

The selection of these models is justified, as they elucidate the mechanism of drug release. In our case, the release profiles correspond to the first-order model, particularly for the unfilled PVA cryogels (Table 3). High values of the coefficient of determination for the Higuchi model (Table 3) indicate that drug release predominantly exhibits a diffusion character according to the Fick’s law. The values of the release rate index n from the Peppas–Korsmeyer model for all studied PVA cryogel samples were less than 0.45 (Table 3), further confirming that the release mechanism is driven by Fickian diffusion. Other release mechanisms are minimally impactful in this scenario, as the cryogel samples are already in a swollen state, allowing for only slight a relaxation of the polymer chains, with no observed dissolution processes or erosion of PHB microspheres. The kinetic constant K from the Peppas–Korsmeyer model suggests a decreased drug release rate in the presence of PHB microspheres in the PVA cryogel matrix, particularly evident with MS-2 particles.

To determine the diffusion coefficients of the drug in the carrier matrix, we used the equation derived from Fick’s second law for cylindrical samples [56,58,59]:

M_t_/M_∞_ = √(4D/πR^2^)√t, (4)

where M_t_ is the amount of drug released at the time t, M_∞_ is the drug amount released at the infinite time, D is the diffusion coefficient of drug in the carrier matrix, and R is the radius of the sample.

Considering that the Higuchi model (2) is based on the Fick’s law, then

k_h_ = √(4D/πR^2^), (5)

From here, the diffusion coefficients (D) of the ibuprofen sodium salt were calculated (the radius of the samples was 0.75 cm):
Unfilled PVA Cryogel1.81 × 10^−5^ cm^2^/sPVA cryogel filled with MS-1 particles1.64 × 10^−5^ cm^2^/s PVA cryogel filled with MS-2 particles1.58 × 10^−5^ cm^2^/s

The study of the release kinetics of ibuprofen sodium salt from both unfilled PVA cryogel samples and composite PVA cryogels filled with PHB microspheres showed that the slowest release occurred from the samples containing MS-2. The size, distribution, and density of microspheres per unit volume of cryogel play a critical role in modulating the release rate by enhancing the interaction between the drug and filler particles and prolonging the release from the composite cryogel matrix.

To further elucidate the release kinetics of ibuprofen sodium salt from the PVA cryogel samples, we employed the Weibull function (6), a well-established tool for analyzing drug release mechanisms from various polymer matrices, which allows the assessment of the influence of the matrix on the drug release [55,60,61,62,63].

M_t_/M_∞_ = 1 − exp(−a × t^b^), (6)

where M_t_/M_∞_ is the fraction of dissolved matter released from the matrix during time t; parameters a and b are constants.

The constant b is generally regarded as a descriptor of the gel matrix structure, serving as an indicator of the release kinetics related to the matrix characteristics [62]. A b value less than 0.35 indicates a highly disordered space, which is significantly different from a percolation cluster. When the matrix has a fractal cluster structure, b values fall between 0.35 and 0.39. If b is between 0.39 and 0.69, diffusion takes place within a fractal or disordered substrate that is separate from the percolation cluster. In contrast, a b value of 0.69 to 0.75 suggests a clearly defined geometric (Euclidean) matrix structure [62].

**Figure 9:**
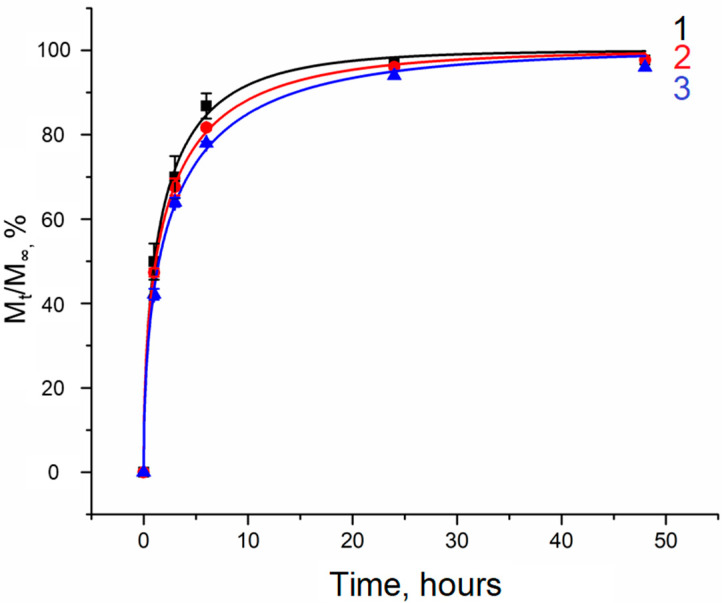
Weibull model plot for the release of ibuprofen sodium salt. Curve 1—cryogel sample without filler; curve 2—cryogel sample with the addition of porous PHB microspheres; curve 3—cryogel sample with the addition of non-porous PHB microspheres.

The analysis of Na-IBPF release from PVA cryogels (Figure 9) via the Weibull function corroborates the conclusions derived from earlier models. The sodium salt of ibuprofen is released most rapidly from the samples without microspheres, whereas the presence of PHB microparticles exerts a prolonging effect on the release process. This conclusion is supported by the coefficient a (Table 4), representing the release rate constant [62]. The parameter b for all samples falls within the range of 0.39–0.69, suggesting that diffusion transpires in a disordered substrate. Furthermore, while b describes the matrix structure, it can also indicate interactions between the gel matrix and the releasing agent, with higher values of b reflecting weaker interactions [64]. Notably, the highest b values correspond to samples lacking PHB microspheres (Table 4). Consequently, the presence of spherical PHB particles in the PVA cryogel matrix contributes to slowing down the release of ibuprofen sodium salt, which is attributed to drug adsorption on the surface of the microspheres; thus, a greater number of microspheres per unit volume of cryogel correlates with increased drug adsorption.

Due to the pronounced “burst” effect (Figure 9) [65], which is observable within the first 6 h of release and attributed to the high solubility of the drug in water, and the presence of a network of interconnected macropores in cryogels, these materials may be particularly beneficial in scenarios requiring rapid drug delivery to the target.

### 2.5. Simvastatin Release

Simultaneously with the formation of PHB microspheres, simvastatin was introduced into the system at a concentration of 4.35 mg per gram of the polymer. Spectrophotometric monitoring of the process revealed that 1 g of dry MS-1 contained 3.76 ± 0.74 mg of simvastatin (~86%), while dry MS-2 contained 4.17 ± 0.57 mg (~96%). The observed loss of the drug compared to the initial amount is likely attributable to partial leaching during various process stages, such as preparation of the primary emulsion, solidification of PHB through chloroform evaporation from the microdroplet phase, and subsequent rinsing of the resultant microspheres with water. Reference samples of PVA cryogels without PHB microspheres were loaded with a comparable amount of simvastatin (~4.17 mg) by dissolving it in the initial aqueous solution of PVA.

**Figure 10:**
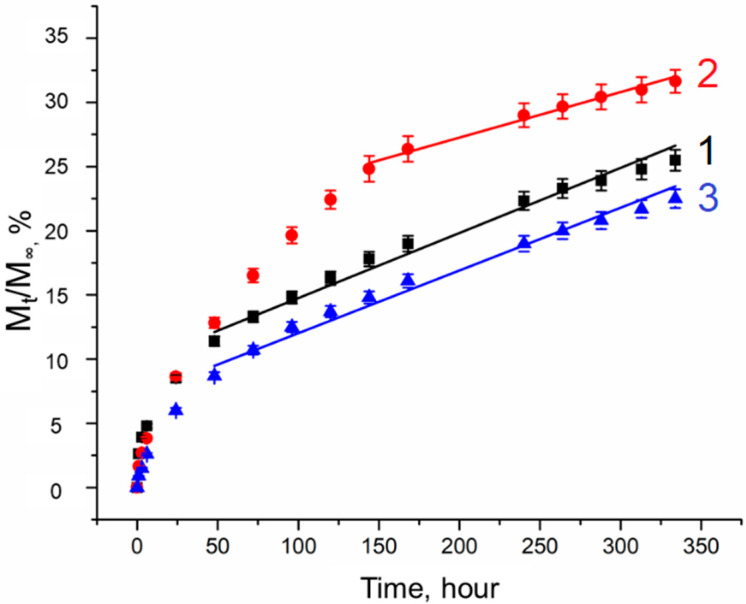
Simvastatin release. Curve 1—cryogel sample without filler; curve 2—cryogel sample with added porous PHB microspheres; curve 3—cryogel sample with added non-porous PHB microspheres.

The kinetics of simvastatin release from composite cryogels containing MS-1 and MS-2 was investigated using unfilled PVA cryogels as control samples (Figure 10). The release profiles were analyzed using the Higuchi (2), Peppas–Korsmeyer (3), and zero-order models (7).

M_t_/M_∞_ = k_0_t, (7)

where t is time, M_t_ is the amount of drug released at the time t, M_∞_ is the drug amount released at the infinite time, and k_0_ is a constant of apparent velocity of dissolution [61,62].

The release profiles aligned more closely with the zero-order model (Table 5), except for the first hours of the release, which exhibited a minor “burst” effect. This “burst” effect is likely attributable to the fact that during the preparation of microsphere suspensions in PVA solutions, some amount of simvastatin could diffuse into the cryogel matrix. In the case of unfilled cryogels, the “burst” effect is related to the reduction in the diffusion path that the compound must pass to exit the cryogel. High values of the R^2^ coefficients obtained from the Higuchi model (Table 5) indicate that the drug release process is primarily diffusion-based, adhering to Fick’s law. For all studied samples, n ≤ 0.45 (Table 5) testifies that the release mechanism is predominantly governed by Fickian diffusion.

Using Equations (4) and (5), the diffusion coefficients of simvastatin were determined (the radius of the samples was 0.75 cm):
Unfilled PVA Cryogel2.58 × 10^−8^ cm^2^/sPVA cryogel filled with MS-1 particles4.24 × 10^−8^ cm^2^/s PVA cryogel filled with MS-2 particles1.85 × 10^−8^ cm^2^/s

The data indicate that the release of simvastatin from both unfilled and composite PVA cryogels is characterized by a prolonged release profile, with no more than 30% of the substance released over a period of 350 h. The slowest release of simvastatin occurred from the samples containing MS-2. This difference in the kinetics of simvastatin release from samples with PHB microspheres of varying porosity can be explained by the necessity for drug molecules to diffuse through the microsphere phase first. In the case of MS-2, this process occurs at a significantly slower rate than with MS-1, as indirectly confirmed by the values of the kinetic constant K (Table 5), which reflects the structural and geometric characteristics of the matrix as well as the release rate.

When dealing with simvastatin, which is poorly soluble in water, the absence of a pronounced “burst” effect is evident (Figure 10), with approximately 5% of the drug being released in the first hours. Subsequently, the release profile is more consistent with a monophasic release for both unfilled cryogels and composite cryogels containing MS-2, suggesting that a steady-state concentration of the drug can be maintained within the therapeutic range over an extended duration.

It should be noted that that the degradation of PHB particles, which may influence simvastatin release, occurs over a significantly extended timeframe (~6–10 weeks) [66]; by this point, the release of simvastatin is presumed to have ceased.

The reasons for the faster release of simvastatin from MS-1 cryogels compared to other samples remain to be elucidated, with further investigation necessary to uncover the precise mechanism.

## 3. Conclusions

Macroporous physically cross-linked PVA cryogels, both unfilled and composite, represent a significant advancement in drug delivery systems. The focus of this study was on composite PVA cryogels filled with spherical, dispersed poly(3-hydroxybutyric acid) particles both with and without macroscopic pores. The size and morphology of the obtained microspheres were evaluated, as well as their impact on the physical and mechanical properties of PVA composite cryogels. It was found that PHB particles without macroscopic pores, due to their smaller size and increased contact area with the PVA cryogel matrix, have a more pronounced effect on enhancing the elastic modulus of the composite cryogel samples. The kinetics of Na-IBPF and simvastatin release from PVA cryogels, comparing those with and without PHB microspheres, were studied. The findings demonstrate that the incorporation of spherical PHB particles in PVA cryogels, particularly MS-2, prolongs the release of the drugs investigated due to their interaction with the dispersed phase. Thus, these materials hold promise as effective delivery systems for both soluble and poorly water-soluble drugs.

## 4. Materials and Methods

### 4.1. Materials

The following substances were used without additional purification: polyvinyl alcohol with molecular weight of 86,000 Da and a 100% deacetylation degree (Acros Organics, Belgium), poly(vinyl alcohol) (PVAs) with molecular weight of ~60 kDa and an 89% deacetylation degree (18/11 trade mark: PO “Azot”, former USSR), ibuprofen sodium salt (Sigma Aldrich, USA), chloroform (reagent grade) (Komponent-Reactiv, Russian Federation), ammonium carbonate (reagent grade) (Khimmed, Russian Federation), diethyl ether (reagent grade) (Khimreaktiv, Russian Federation), and deionized water.

SVN was isolated from the milled pellets of the commercial drug, “Simvastatin” (Ozon Pharm LLC, Russian Federation), via extraction with chloroform. The resulting solution was filtered, and then simvastatin was precipitated with diethyl ether, separated by filtration, and air-dried. The purity of the resultant SVN was confirmed using the TLC analysis.

Poly(3-hydroxybutyric acid) (Biomer, Germany) with molecular weight of ~300 kDa (capillary viscometry measurements) after additional purification by the dissolution of the commercial polymer in chloroform was used, followed by the PHB precipitation in an excess isopropyl alcohol, filtration, and drying.

### 4.2. Methods

#### 4.2.1. Unloaded and SVN-Loaded PHB Microspheres

Two types of PHB-based microspheres, namely those containing macroscopic pores, i.e., MS-1, and the microspheres without macroscopic pores—MS-2—were fabricated as described elsewhere [41]. In brief, in order to prepare MS-1 particles, 12 mL of 45 mg/mL PHB chloroform solution were intensively mixed with 6.6 mL of aqueous 50 mg/mL ammonium carbonate solution and then added to 210 mL of aqueous 10 mg/mL solution of PVAs with intense stirring at 750 rpm using an RZR 1 stirrer (Heidolph, Germany). Subsequent stirring was continued until almost complete evaporation of chloroform, followed by separation of the formed PHB microspheres, which were then filtered-off and rinsed with distilled water (7 × 30 mL). Upon the preparation of MS-2 samples, the stage of mixing with ammonium carbonate solution was omitted. The microspheres of both types thus obtained were entrapped into the PVA cryogels for further use in the physico-mechanical tests. In turn, upon the preparation of drug-loaded PHB microspheres, simvastatin was dissolved in the concentration of 2.25 mg/mL in the PHB chloroform solution, and subsequent operations were the same as during the fabrication of unloaded microspheres. Both types of microspheres were visualized using an optical stereomicroscope, SMZ1000 (Nikon, Tokyo, Japan), equipped with a digital image registration system, MMC-50C-M (MMCSoft, Russian Federation). The particles’ sizes were evaluated by analyzing the obtained images using Image J (version 1.54k) program (National Institutes of Health, USA).

#### 4.2.2. Microstructure of PHB Microspheres

SEM images of dry PHB microspheres pre-sprayed with gold (ion sputtering device IB3, Giko, Japan) were obtained using a scanning electron microscope JSM-6380LA (JEOL Ltd., Kyoto, Japan), and the accelerating voltage was 20 kV (SEI mode).

#### 4.2.3. Filler-Free PVA Cryogels and Composite PVA/PHB Cryogels

Aqueous solutions of PVA with polymer concentration ranging from 73 to 138 g/L were prepared according to the procedure published earlier [67,68,69]. For this purpose, a known amount of dry polymer powder was dispersed in the required volume of water, and such suspension was incubated for 18 h at room temperature to swell the polymer, followed by heating for 1 h on a boiling-water bath with stirring until the PVA was completely dissolved. The sample was weighed before and after heating, and the amount of evaporated water was compensated after the solution was cooled to room temperature. The resultant solutions were then used for the preparation of PVACGs.

When it was necessary to fabricate the PHB-filled cryogels, the amount of water in the wet PHB microspheres was also taken into account upon the preparation of their suspensions in the respective PVA solutions. Further on, such suspensions were subjected to a freeze/thaw process, thus resulting in the composite PVA/PHB cryogels.

To form cryogels, PVA solutions and suspensions of PHB microspheres in PVA solutions were poured into the duralumin cylindrical molds (inner diameter 15 mm, height 10 mm) [69,70,71,72], which were placed in the chamber of a precision programmable cryostat Proline RP 1840 (Lauda, Konigshofen, Germany), where the samples were frozen at −20 °C and incubated for 12 h. The temperature was then increased to 20 °C at a rate of 0.03 °C/min controlled by the microprocessor of the cryostat in order to thaw the frozen samples, thus giving rise to the target unfilled and composite PVA cryogels that were either unloaded with a drug or contained filler PHB–microspheres loaded with SVN.

#### 4.2.4. Physico-Mechanical Properties of Filler-Free and Composite PVACGs

Young’s compression modulus (E) values of cryogel samples were found based on the linear part of the stress–strain relationships recorded with an automatic texture analyzer TA-Plus (Lloyd Instruments, UK) in the uniaxial compression mode, as described previously [34]. The loading rate was 0.2 mm/min; the tests were carried out at a strain rate of up to 30%. E values were measured for three parallel specimens; the specimens were prepared in 3–5 independent experiments. The results thus obtained were averaged.

#### 4.2.5. Saturation of PVACGs with Ibuprofen Sodium Salt

To load the filler-free composite PVA cryogels with Na-IBPF, each cylindrical cryogel sample was placed in a vial with 5 mL of 1 mg/mL aqueous solution of this model drug. Then, UV/VIS spectra of the liquid phase were recorded using a T70 UV/VIS Spectrophotometer (PG Instruments Ltd., Wibtoft, UK) at intervals of 24 h for 5 days, reaching the equilibrium state of the dissolved substance distribution between the gel and liquid phases.

#### 4.2.6. Kinetics of Drugs Release from the PVACG-Based Carriers

(i) SVN: Each of the cylindrical SVN-containing cryogel samples (their volume was ~2.1 mL) fabricated according to Section 4.2.3 was immersed in 5 mL of 0.05 M Na–phosphate-buffered solution (pH 7.4) and incubated at room temperature for required time intervals. These cryogel samples initially contained simvastatin in an amount of 1.162 ± 0.230 mg per a sample. UV spectra of the respective supernatants were then recorded using the T70 UV/VIS Spectrometer (PG Instruments Ltd., UK), after which the outer liquid around the PVA composite cryogel was replaced for a fresh 5 mL portion of the buffer solution. The same operations were repeated for the required number of cycles. The solute content in the liquid phase was found based on the preliminarily constructed calibration dependence of UV-absorbance at 238 nm on the SVN concentration.

(ii) Na-IBPF: The employed procedure was analogous to the previous case, (i) except for the wavelength used for measurements of UV absorbance, which, for Na-IBPF, was equal to 264 nm.

In the drug release studies, 3 samples were used for each type of cryogels in a series and then replicated them under similar conditions.

## Figures and Tables

**Table 1:** The values of the particle size and the Gaussian function coefficients for the PHB microspheres.

	MS-1	MS-2
µ	299	75
σ	101.45	35.26
R^2^	0.99	0.99

**Table 2:** Composition of initial solutions and suspensions used for the preparation of the filler-free and composite PVA cryogels.

Sample	PVA Concentration, g/L	PHB Concentration (MS-1), g/L	PHB Concentration (MS-2), g/L
1	72.6	—	—
1a	72.6	11.4	—
1b	72.6	—	11.4
2	100	—	—
2a	100	11.4	—
2b	100	—	11.4

**Table 3:** Coefficient values for the first-order, Higuchi, and Peppas–Korsmeyer models.

Type of Drug Carrier	Frist Order		Higuchi		Peppas–Korsmeyer		
	k_1_	R^2^	k_h_	R^2^	n	K	R^2^
Unfilled PVA Cryogel	−0.108	0.94	0.38	0.95	0.30	0.50	1
PVA cryogel filled with MS-1 particles	−0.106	0.92	0.36	0.95	0.30	0.48	0.99
PVA cryogel filled with MS-2 particles	−0.13	0.90	0.35	0.98	0.33	0.43	0.99

**Table 4:** The values of the coefficients for the Weibull model.

Type of Drug Carrier	a	b	R^2^
Unfilled PVA Cryogel	0.68	0.56	0.99
PVA cryogel filled with MS-1 particles	0.64	0.53	0.99
PVA cryogel filled with MS-2 particles	0.56	0.52	0.99

**Table 5:** Coefficient values for zero-order, Higuchi, and Peppas–Korsmeyer models.

Type of Drug Carrier	Zero Order		Higuchi		Peppas–Korsmeyer		
	K_0_	R^2^	k_h_	R^2^	n	K	R^2^
Unfilled PVA Cryogel	0.0006	0.93	0.0145	0.99	0.41	0.022	0.99
PVA cryogel filled with MS-1 particles	0.0009	0.89	0.0186	0.99	0.45	0.023	0.98
PVA cryogel filled with MS-2 particles	0.0006	0.93	0.0123	0.99	0.45	0.012	0.99

## Data Availability

The original contributions presented in the study are included in the article, further inquiries can be directed to the corresponding author.
